# Quantification: The View From Natural Language Generation

**DOI:** 10.3389/frai.2021.627177

**Published:** 2021-05-17

**Authors:** Kai-Uwe Carstensen

**Affiliations:** Department of German Linguistics, School of Arts and Humanities, University of Siegen, Siegen, Germany

**Keywords:** language and computation, quantification, semantics, generation, plurality, collectivity, cumulativity, distributivity

## Abstract

Quantification is one of the central topics in language and computation, and the interplay of collectivity, distributivity, cumulativity, and plurality is at the heart of the semantics of quantification expressions. However, its aspects are usually discussed piecemeal, distributed, and only from an interpretative perspective with selected linguistic examples, often blurring the overall picture. In this article, quantification phenomena are investigated from the perspective of natural language generation. Starting with a small-scale, but realistic scenario, the necessary steps toward generating quantifier expressions for a perceived situation are explained. Together with the automatically generated descriptions of the scenario, the observations made are shown to present new insights into the interplay, and the semantics of quantification expressions and plurals, in general. The results highlight the importance of taking different points of view in the field of language and computation.

## 1 Introduction

At the end of the 1970s, a survey of knowledge representation methods in AI based on a questionnaire uncovered an enormous diversity and led to quite unflattering opinions: “as one said, ‘Standard practice in the representation of knowledge is the scandal of AI’” (Newell, 1982, p. 92). For many, logic was the resort out of this situation, even if only at the knowledge level [but, cf. McDermott’s *Critique of Pure Reason* and its discussion in Computational Intelligence, 3 (1987)]. In every relevant formal language beyond propositional logic, this inevitably involves *quantification* as a central part. However, formal logics are quite restricted when compared to the expressability of quantification aspects in natural language, and both logicians and computationalists have a keen interest in linguistic semantics’ progress in that matter. Unfortunately, as shown below, a corresponding survey in this field would yield no less diversity than the one above, and standard practice in today’s treatment of quantification in linguistics and natural language processing, as well as its slow progress, could well be regarded as a “scandal of language and computation”. The following elaborates on this argumentation, but offers a perspective shift as part of a solution.

Typically, linguistic semantics is interpretative. That is, most corresponding work is based on some linguistic data, focuses on a few phenomena, and presents proposals of how these phenomena, given the data, can be analyzed. The analyses, in turn, are based on formal languages interpreted with formal models that represent the relevant states of affair as given and only as far as needed for the analyses. As common as it is, this interpretation perspective disregards the fact that every linguistic datum presupposes an act of language production/generation and, hence, the primacy of that stage (although this is ultimately a chicken-and-egg question, of course).

The existence of different views on language is an old linguistic insight, reflected, for example, in the “semasiology”/“onomasiology” dichotomy. Its importance has been especially recognized in practical natural language generation research, where the focus is on the contrast to natural language comprehension: “Existing comprehension systems as a rule extract considerably less information from a text than a generator must appreciate in generating one” (McDonald, 1993). As a rule of thumb, the generationist is interested in covering the *range* of phenomena in some domain (to prevent non-applicability in some new scenario, i.e., nonbrittleness of the system), while the interpretationist often is content with presenting an elaborate theory accounting for a restricted set of phenomena. In the present context, the interpretationist would ask for (the possibility of) certain readings of sentences containing quantifier expressions, and the generationist would be interested in the variation of expressions verbalizing a given scenario.

Nirenburg and Raskin (2004) argued that “linguistic theories profess to strive to produce complete descriptions of all the data in their purview [… but that in practice, … ] corners are cut” (p. 57). They also cited Bar-Hillel having “criticized the methodology of logical semanticists: they unduly constrain their purview, and within that limited purview, concentrate primarily on exceptions” (p. 360). Theoretically, therefore, the interpretation perspective may lead to a bias, or worse, to wrong analyses, if aspects evident from the generation perspective are disregarded. In this article, I want to show that this is actually the case for the semantics of quantification expressions and plurals, and that the generation view offers an effective alternative for the treatment of quantification phenomena.

In the following, I will first summarize the main ideas of quantifiers and quantification in modern semantics relevant for the present purposes, along with some of the problems concerning the interplay of collectivity, distributivity, cumulativity, and plurality. After that, I will apply the generation perspective by demonstrating how (sentences containing) quantifier expressions can be automatically generated for a realistic scenario, exemplifying an improved scheme for the interplay. The discussion of the observations made and the small-scale proof-of-concept implementation will provide evidence for the necessity of re-viewing the semantics of quantification expressions and plurals.

## 2 Aspects of Quantifiers and Quantification

In two respects relevant here, the work of Frege can be regarded as the starting point both of modern logic and formal semantics: first, by shaping what has evolved into first-order predicate logic (FOPL), and second, in the idea of semantic compositionality later realized by the use of the (typed) lambda calculus.^1^ With regard to the invention of predicate logic, Peters and Westerståhl wrote: “One crucial addition in the new logic was variable-binding: the idea of variables that could be bound by certain operators, in this case the universal and existential quantifiers” (Peters and Westerståhl, 2006, p. 34).

Successful as it has been in the past century, FOPL is quite restricted: with ∀ and ∃, it only has two quantifying operators, and the variables range over flat domains of *individuals*. Correspondingly, “[s]everal kinds of constructions, sentences, and inferences that cannot be symbolized in first-order logic are known. Perhaps, the best-known of these involve numerical quantifiers such as ‘more,’ ‘most,’ and ‘as many’” (Boolos, 1984, p. 431). Unfortunately, the “crucial” aspect of ‘quantification as variable-binding’ can also be regarded as the central source of confusion, as it confounds at least aspects of variable-binding, existence, quantification, distribution, and scope.

Furthermore, important aspects of quantification such as the explicit distribution and accumulation of pluralities or the proper treatment of collective predication are outside the representational scope of FOPL. For instance, neither does ∀ capture the distinction of *for all [men]* and *each [man]* (necessary for the exclusion of *Each man meets) nor is it suited to bind an argument variable of a collective predicate such as *meet* at all.

Based on the insight that natural language quantification must be treated on a different formal level, Montague introduced a relational view of quantifiers (later called *generalized quantifiers* in *Generalized Quantifier Theory* (GQT), see Peters and Westerståhl, 2006). According to that view, quantifiers have to be treated as determiners that relate two properties: restrictor (noun phrase meaning) and scope. Quantifiers could then be regarded as imposing a certain condition on the intersection of their denotations/sets [see (1)].

(1) $〚\left(Generalized\right)Quantifier〛=\lambda R\lambda S\left[Condition_{Quantifier}\left(R,S\right)\right].$

Montague has become famous for showing that natural language can be given a straightforward compositional semantic treatment with such a scheme (see Montague, 1973). Yet, there are quite a number of arguments against treating quantifiers wholistically as determiners (cf. Krifka, 1999; Szabolcsi, 2010, in general). A particular problem concerns the observation that the GQT scheme is only applicable down to some level of linguistic granularity. It disregards compositionality aspects of complex quantifiers, and neither explains why *almost a/some/many/… are not well-formed expressions nor reflects the observation that, for example, *almost* behaves exactly as in the adjectival domain (*almost as long as*). It ignores the fact that there are striking structural analogies between the domains of quantification and gradation (see (2) for a comparison, and Carstensen 2013, on gradation), and it led to treating both sets of phenomena differently.

(2) (*almost) how many - (*almost) how high(almost) as many - (almost) as high(*almost) more/less than - (*almost) higher/lower than(almost) most people - (almost) the highest tower/glass(*almost) many people - (*almost) high tower/glass(almost) ten people - (almost) ten meters high tower/glass(almost) all people - (almost) full glass(almost) no people - (almost) empty glass(*almost) some people - (*almost) slightly full/dirty glass

The congruency in (2) has been scarcely recognized so far, which may be traced by the fact that the semantic phenomena are nonoverlapping for the most part: while classic quantification deals with the upper and lower ends of the quantity scale (*all, no*) and with existence (*a, some*), these aspects are out of focus in typical relative adjectives. As can be seen from (2), however, gradation phenomena are analogous to the full range of quantification phenomena, especially as there are adjectives [the so-called absolute adjectives such as *full*, *empty*, *dry*, and *wet*, see Kennedy (2007)] that also involve reference to scale boundaries. Accordingly, this opts for a more fine-grained compositional treatment of quantifiers compatible with the semantics of gradation.

The compositionality of a sentence with *multiple* quantifiers is tricky in itself [see the meaning of *give* in (3), adapted from Blackburn and Bos (2005)], and handling their *scope* has been a persistent topic for decades. Starting with specific procedural methods (by Montague and others), the problem turned declarative with the mechanisms of underspecification developed in the 1990s (see Reyle, 1993). While GQT already requires the full power of the lambda calculus for compositionality (instead of some simpler, flat compositional scheme), this has led to (too) powerful mechanisms that often generate too many scope readings and at the same time do not explain observable *asymmetries* in actual orderings of two quantifiers: “These asymmetries present a challenge to all frameworks that attempt to capture scope phenomena in terms of uniform operations over generalized quantifiers […]” (Steedman, 2012, p. 29).

(3) $〚give〛=\lambda Q\lambda P\lambda x\left[P\left(\lambda y.Q\left(\lambda z.give^{\prime }\left(x,z,y\right)\right)\right)\right].$

One example for this is the contrast in (4) (taken from Sæbø, 1995), where (4a) shows scopal ambiguity, while (4b) does not. Steedman’s example in (5) shows that while there may be only three kissed girls altogether (in a wide-scope reading of the girls-NP), there are no varying halves of the boys.

(4)
  a. Some nurses are always on duty.
  b. There are always some nurses on duty.
(5) Exactly half the boys in the class kissed three girls.

Dynamic semantics approaches following Montague adopted the relational treatment of quantifiers and rather shifted the view from sentence compositionality to discourse compositionality (see, e.g., the discourse representation theory (DRT) of Kamp and Reyle, 1993), also introducing explicit underspecified structures for (scopal) ambiguities. However, especially when looking at larger linguistic units (whole texts), it becomes apparent that it is more adequate to exploit underlying principles of representation and inference as implicit disambiguation strategies (e.g., presupposition justification and accommodation, cf. Carstensen, 2000) than to try dealing with the rising number of procedural options or the growing complexity of underspecification structures. Nouwen concludingly writes about the GQT-style quantifiers: “the GQT notion of a quantifier is not really very suitable if we want to learn more about the semantics of expressions of quantity” [(Nouwen, 2010, p. 254)].

In the 1980s, at the latest, it became clear that *plurality* should better be modeled with *pluralities* (*plural entities*). This involves either elements of the powerset of a domain of individuals (Winter, 2002) or *sums* of individuals (Link, 1983)^2^. Using Link’s “*”-operator, the impact of grammatical plural can then be represented as pluralizing a flat domain of individuals by adding sums of them as in (6) (see Nouwen, 2014).

(6) 〚boys〛=∗〚boy〛

With plural entities, *collectivity* can be modeled directly. For example, in *Three boys eat a pizza*, there might only be one pizza, eaten by the collection of three boys (which corresponds to the “referential” reading of *Three boys*). Collectivity is also present in collective verbs such as *meet*, where the predicate’s argument is necessarily nonindividual.

In FOPL, the collective pizza-eating interpretations (e.g., the boys jointly munching pieces of a set of three pizzas) or *cumulative* ones (according to which there are eating events with boy-eaters and pizza-eatees whose numbers sum up to three, respectively) are not available at all. This is different with *distributivity*. For example, in *Every boy eats a pizza*, left-to-right interpretation of a standard-order formula (starting with ∀xϕ) directly leads to the correct result. Yet, if that scheme were applicable for other quantifiers in FOPL, the sentence *Three boys eat three pizzas* would only receive distributive interpretations (either each of the boys eating three (different) pizzas or, less likely to get, each of the three pizzas being eaten by three (different) boys).

A common approach in modern semantics to represent distributivity is the operator DIST in (7) (see Nouwen, 2014) that asserts the application of property *P* to all atomic parts *ß* of plurality *α*. It can occur as a covert operator or represent the contribution of *each* in examples such as (8).

(7) DIST=λP.λα.∀β≤α[Atom(β)→P(β)].
(8)
  a. Three boys have eaten a pizza (covert).
  b. Each boy has eaten a pizza (prenominal).
  c. Each of the three boys has eaten a pizza (DIST + partitive NP).
  d. Three boys each have eaten a pizza (post-nominal).
  e. Three boys have each eaten a pizza (floating).
  f. Three boys have eaten one pizza each (binominal, see Safir and Stowell, 1988).

As has been discussed in Scha and Stallard (1988), distributive predication may be “partial” if predication to a collection is distributed to nonatomic parts of that collection, involving a collective verb (as in *Three boys eat a pizza*, where two boys jointly eat a pizza, the third one eating a pizza alone, or as in *the juries and the committees gathered*, where there can be more than one gathering). To account for these data, there exist proposals for the distribution operator (see Nouwen, 2014) that represent distribution of a predicate’s application *to relevant parts of a plurality minimally covering it* as in (9).

(9) $DIST_{C}\left(P\right)=\lambda x\forall y\in C_{x}\left[P\left(y\right)\right]$, where $C_{x}$ is some pragmatically determined minimal cover of *x*

Unfortunately, such an operator is too general. With respect to the example (10) ( (35a) in his work), Nouwen pleads for possible different subcollections of eggs, each costing € 2. Assume, however, a scenario where there are three of them (sizes 1, 2, and 3). It does not seem to be describable distributively by (10).

(10) These six eggs cost € 2.

*Cumulativity* can be characterized as the phenomenon of (a) plurality, when collectivity and distributivity do not apply, as in the famous example of Scha (1984) in (11). This is deliberately vague because cumulativity has received widely differing treatments (from simple denial in favor of collectivity analyses via lexical analysis to pluralization of (verbal) predicate accounts^3^). Most assume the necessity of a symmetric non-scopal relation (to capture examples like (11)), and some restrict cumulativity to relations of individuals, while others allow cover readings [cf. Beck and Sauerland (2000) and the discussions in Nouwen (2014) and Champollion (to appear)]. Classically, it is NPs that are considered in theories of scope and plurality (and are controversially discussed, see, e.g., Krifka, 1992). Especially for cumulativity, the role of *events* is increasingly judged as important (see Landman, 1996 for an overview).

(11) 600 Dutch firms have 5,000 American computers.

As to the problem of the *interplay* of quantification aspects, consider a simple scenario representable as a reciprocal hate relation of pairs of individual boys (in total: three) and girls (in total: four), each girl only hating one boy, and only one boy hating two girls. With the present means of (linguistic) quantification, it is hardly possible to reflect this constellation: in (12a), collective, distributive, or cumulative readings cannot be excluded, and in (12b), the typical reading is over-distributive, as quantifiers have to be *linearly ordered* in standard formalization. The desire to have *partially ordered* quantifiers has led to the concept of *branching quantifiers* (with Hintikka’s famous linguistic example (13)), but as Sher (1990) shows, it is still hard to prevent over-distributivity with standard logical means that cannot cope with cumulativity without distributivity.

(12)
  a. Three boys hate four girls {and vice versa /reciprocally/ four girls hate three boys}.
  b. Each of three boys and each of four girls hate each other.
(13) Some relative of each villager and some relative of each townsman hate each other.

There is a different, weaker conception of cumulativity, however, that simply refers to the accumulation of argument instances due to different *events* (therefore, noncollective). It can be the converse perspective of the distributive case in (14a), and it can occur in a distributive context [(14b), where the details of the eating events are glossed over].^4^ In the former, the subject varies with the event, and in the latter, the object varies. Based on such considerations, there are some who include *plural events* in theories of plurality (see Landman, 1996 for an overview) to cope with the interplay of quantification aspects. This is problematic, however, because while “nominal” entities can be pluralized (*three boys, many times,* etc.), events cannot (*Peter jumps three/*Peter three jumps) (cf. also Carstensen, 2011 on this point).

In both cases of (14), there is no symmetric relation available, and Champollion (to appear) discusses other examples in which cumulativity and distributivity interact, which he says is “surprising on many formal accounts”. In the following, I will show that such an interaction ((10) being another possible case in point) is, on the contrary, not surprising at all, and an essential ingredient will be the systematic consideration of events for theories of quantification.

(14)
  a. Few disagreed.
  b. Every boy ate (on the whole/all in all/in total) three pizzas.

Summing up, current formal semantics presents a fragmented, incoherent picture and insufficient treatment of quantification that rests solely on compositionality and more or less complex domains. Yet, with an overly powerful lambda calculus and relational quantifiers, it over-generates scope readings, and with its simple ontology, it cannot even distinguish collections and groups as different plural entities. On the whole, it does not account for the complex interplay of collectivity, distributivity, cumulativity, and plurality in the semantics of quantification expressions.

## 3 The Generation Perspective

### 3.1 Preliminaries

Taking the generation perspective requires some preliminary considerations and clarifications. First, there is a rough distinction in the language generation literature between *what* to say (content determination or macroplanning) and *how* to say it (which is divided into structuring the content, or microplanning, and grammatically *realizing* it). In general, the starting point of generation is an underlying question (or *quaestio*) in some context. Considering quantification, one quickly realizes that scenarios for constellations with classical quantifiers are either rare (e.g., *Every pope knows every apostle*) or uninteresting (*Every man has a mother*), and that one should select specific scenarios to elicit interesting verbalizations. For the purposes of this article, I will use the model of a classical TRANSFER scene, and the quaestio will be “what happens?” with a certain perspective. The focus will therefore be on the microplanning task selecting quantified NPs, disregarding the realization part.

Technically, I will use simple (computational linguistics) methods for the present investigation. As the focus is on linguistic questions of quantifier semantics (as opposed to questions of computational linguistic theories, methods, or implementation), they are stripped down to the bare bone of relevant distinctions.^5^ This is done by using pure PROLOG (PROgramming in LOGic, Clocksin and Mellish, 1981; the environment I use is SWI-Prolog).

Semantic models can be directly represented in PROLOG as *facts* (quantifier-free atomic FOPL formulas delimited with “.”) of its *database* (the so-called *knowledge base*). For example, prop(“class”,“boy(s)”,b1) represents the fact that there is some boy b1 [i.e., b1 is of class “boy(s)”]. Therefore, the set of X such that prop(“class”,“boy(s)”,X) is true (i.e., is in the knowledge base) corresponds to the denotation of *boy* in the interpretative perspective.^6^ Assuming that the scenario is about three boys exchanging various things with four girls (one other girl, g12, is not involved), the representation of those entities is as follows:

prop(“class”,  “boy(s)”, b1). prop(“class”, “boy(s)”, b2).

prop(“class”,  “boy(s)”, b3). prop(“class”,   “girl(s)”, g1).

prop(“class”,   “girl(s)”, g11). prop(“class”, “girl(s)”, g12).

prop(“class”,   “girl(s)”, g2). prop(“class”, “girl(s)”, g3).

prop(“class”,   “tulip”, t1). prop(“class”, “rose”, t11).

prop(“class”, “chocolate bar(s)”, t2). prop(“class”, “chocolate bar(s)”, t3).

prop(“class”, “gift coupon(s)”, t4). prop(“class”, “gift coupon(s)”, t5).

*Framing* is the process of imposing a perspective on a scene (Fillmore, 1977). In the situational domain, it involves identifying relevant participants in some *order* depending on salience and/or relevance for the speaker (including the identification or attribution of properties such as thematicity and/or agentivity), ultimately verbalizable in the given language. I have greatly reduced the complexity of these aspects by simply representing the ultimate perspective by a verbal predicate that frames the transfer as ‘x giving y to z’ perspective events, as opposed to, for example, ‘z receiving y from x’. This predicate has a referential event argument, following Davidson (1967) (below, I will give further evidence motivating such a Davidsonian approach). Correspondingly, the following PROLOG facts represent the framed scenario to be linguistically described:

give_to(e1,[b1],[t1,t11],[g1]). give_to(e2,[b2],[t2],[g1,g11]).

give_to(e3,[b2],[t3],[g2]). give_to(e4,[b3],[t4],[g3]).

give_to(e5,[b3],[t5],[g3]).

### 3.2 Generating Quantifiers: The Basic Picture

FOPL and GQT are based on either individuals or sets of individuals, respectively. The deliberately construed events of the scenario, however, clearly show that this is not the case. For example, in the event e1, b1 gives two flowers to g1^7^, and likewise, g1 and g11 *collectively* “own” the chocolate bar after e2 happens. Note also that while singular and plural event participant arguments are not categorically distinguished (both are represented alike as sets/collections, see Scha (1984) and the discussion below), the referential event argument is different: it is not only an individual but it also does not give rise to “verbal plurality” (because events cannot be counted, see, e.g., *Peter jumped three/*Peter three jumped). As discussed in Carstensen (2011), this can be explained by the ontological difference between the “verbal” and the “nominal” domain.

**Observation 1.** Event participant argument instances (both singular and plural) are nonindividual (i.e., collections). The referential event argument is an individual.

Observation 1 obviously has repercussions on quantifier semantics, as there can be no simple intersection of sets of individuals.

Given that the speaker has selected a scenario and lexical framing option, how does he generate (scoped) quantification expressions?^8^ Half of the answer has already been given: by exploiting the specified order of the framing, that is, most prominent X-role, less prominent Y-role, and even less prominent Z-role of the chosen verbal predicate (note the difference to a give(…) framing, where the girls would have the second position as indirect objects).^9^ This means first considering the boys, then the flowers, etc., and then, the girls for the description of the scenario. Accordingly, this corresponds to an ordered *accumulation of the respective sets*, determining the scope of the quantification expressions.

**Observation 2.** Cumulativity (in a general, accumulation, sense) is the basic, default phenomenon in quantification.

While such a scheme allows for more framing aspects (e.g., event modifiers and passivization) than the ones considered here, it can be regarded as the source of the *asymmetry* in quantifier scoping noted above: an underlying order based on conceptual framing, to be distinguished from a surface order of the quantification expressions involving possible syntactic rearrangements.

Can the generation process benefit from typical ingredients of compositional semantics? Unfortunately not. Consider a simplified verb denotation such as λzλyλxλe[give(e,x,y,z)]. Evidently, the lambda variables are in a reverse order of the frame roles. One therefore has to distinguish between the semantic representation of *give* and the concept give(e,x,y,z), representing a relation R of giving events (in some context). Rather than being relevant for the process beforehand, the semantic structure is then built as a result of it.

Therefore, it is proposed here that the generation process is basically one involving a sequence of *projections* of R using the ordered variables of the concept (by differentiating the referential variable from the others). A projection $\pi_{i}\left(R\right)$ can be defined as the *i*th projection $\left\{x_{i}|\left(x_{1},\ldots ,x_{k}\right)\in R\right\}$ (i.e., a set) of a *k*-ary relation (Scha, 1984; Kanazawa and Shimada, 2014). The functionality to perform such a projection is provided by the PROLOG predicate *setof*. In the following, the first line is the call of the procedure querying the knowledge base (“accumulate the set of X, where X is the second argument of give_to, as the set/list P”), and the variables are instantiated accordingly in the output below the query.

?- setof(X,give_to(E,X,Y,Z),P).

E = e1, Y = [t1, t11], Z = [g1], P = [[b1]];

E = e2, Y = [t2], Z = [g1, g11], P = [[b2]];

E = e3, Y = [t3], Z = [g2], P = [[b2]];

E = e4, Y = [t4], Z = [g3], P = [[b3]];

E = e5, Y = [t5], Z = [g3], P = [[b3]].

Note, however, that rather than producing the whole set, there are five P-solutions (divided by “;”) because of bound variables. To prevent this, the built-in *setof* allows to existentially bind variables (using the ^-operator) as in the following, giving the desired result, a set of collections:

?- setof(X,E ^ Y ^ Z ^give_to(E,X,Y,Z),P).

P = [[b1], [b2], [b3]].

The library *yall* (standing for *Yet Another Lambda Library*) allows an even more concise query notation by having to specify only the bound variables needed in curly brackets with a “/”-delimiter (here, for Z):

?- setof(Z,{Z}/give_to(E,X,Y,Z),P).

P = [[g1], [g1, g11], [g2], [g3]].

**Observation 3**. The starting point of quantifier generation is projecting elements of R according to the order of the variables in the concept of R.

The main idea of GQT is to base the semantics of quantifier expressions on the intersection of two sets. In the generation perspective, this is different, and the intersection is implicit in the projection. Therefore, quantification does not have to be conceived as relating sets, but can be reduced to measuring the projection set (disregarding aspects of distribution so far).

According to that view, quantification involves a measure function (apparent in questions such as *How many* … *?*) whose degree is qualified by a quantifier. As already shown in (2), quantification in the nominal domain is fully analogous to *gradation* in the adjectival domain. This becomes even more obvious when looking at the inner, compositional structure of quantifier and gradation phrases. As I have shown in Carstensen (2013), one has to distinguish between a degree denoting expression and the phrase with its (possibly empty) head.^10^ This treatment not only allows a straightforward compositional treatment of (*almost*) *every* [see (15)] but can also be generalized to other (numerical) quantifiers [see (*almost/more than*) *three* in (16)].

(15)
  a. $〚every〛\;\;\;\;\;\;\;\;=d_{Q_{\max}}$
  b. $〚\left[\emptyset every\right]〛\;\;\;\;\;\;=\lambda d\left[d\approx d_{Q_{\max}}\right]$
  c. $〚\left[almost\;every\right]〛\;\;\;\;=\lambda d\left[d\;ALMOST\;d_{Q_{\max}}\right].$
(16)
  a. $〚three〛\;\;\;\;\;\;\;\;=d_{3}$
  b. $〚\left[\emptyset \;three\right]〛\;\;\;\;\;\;\;\;=\lambda d\left[d\approx d_{3}\right]$
  c. $〚\left[more\;than/almost\;three\right]〛=\lambda d\left[d\;MORE\_THAN/ALMOST\;d_{3}\right].$

**Observation 4.** Quantifiers denote (sets of) measures of collections. The semantics of quantifier expressions is basically analogous to the semantics of gradation expressions. This also allows quantifier expressions to be realized in/as different parts of speech (as determiners or modifiers).

There is a precondition for this measuring view: it requires a scale common to the elements in question. What is needed, therefore, is “a *generalization* of the distinct entities” [Shaw and McKeown (2000), emphasis in the text] in the projection. This corresponds to finding a complete conceptual cover, that is, a common class, of the projection (allowing expressions such as *every boy /all boys*, as opposed to other possible cover expressions such as *Tom, Dick, and Harry /Harry and two other boys /…*). It is a second step to determine the relevant scale. This can be done *via* the cover class (e.g., to refer to all existing boys as in *all boys are human*; or to a contextually determined subset, e.g., the boys in the scenario, in the common ground of the speaker and hearer) or *via* explicit reference to an established set (*There are three boys. All of them…*). There is presuppositionality in nonnumerical quantifiers (see Heim and Kratzer, 1998), apparent in sentences such as *#Boys gave all presents to girls* (inacceptable if the scenario presents have not been introduced to the hearer).

**Observation 5**. Quantification requires a conceptual cover of (a subcollection of) a single collection (being a such-and-such projection of some relation and being classified so-and-so) so that the collection can be measured along the scale provided by the cover class.

In PROLOG, provided that a recursive get_super predicate is defined, computing the common class is a one-liner: foreach(member(A,Set),get_super("class",C,A)). If there is one according to the represented class hierarchy (aka *ontology*), it will find the most specific superordinate class C of all members of the collection.^11^

It is less clear what this procedural account tells us about declarative semantic representations. For verb semantics, it can be assumed that the collections with their generalization and quantification aspects are lambda-abstracted out of the information cluster, leaving the projection information relating event participant argument and collection variables behind (see (17)); see the discussion for more details on this topic).

(17) 〚give_to〛=λPO λDO λSU $\begin{array}{c} \begin{array}{l} \text{set}\text{of}\left(\text{x,}\left\{\text{x}\right\}/\text{give\_to}\left(\text{e},\text{x},\text{y},\text{z}\right),\text{SU}\right)\\ \text{set}\text{of}\left(\text{y,}\left\{\text{y}\right\}/\text{give\_to}\left(\text{e},\text{x},\text{y},\text{z}\right),\text{DO}\right)\\ \text{set}\text{of}\left(\text{z,}\left\{\text{z}\right\}/\text{give\_to}\left(\text{e},\text{x},\text{y},\text{z}\right),\text{PO}\right) \end{array} \end{array}$

Such an approach is less complex than typical GQT-type ones (see (3)). Evidently, it also allows a more straightforward analysis of nongeneric *bare plurals*: sentences like *Boys give things to girls*, all else being equal, simply lack expression of quantification information.

### 3.3 Distributivity

The basic picture of quantifier generation gets complicated by the fact that the projections of R are not always independent, but sometimes relative to /dependent on another variable’s instances. What is needed, therefore, is to add *selection* to the process of projecting an argument by specifying this variable as bound. There are two ways to view this situation, roughly corresponding to the distinction of recursivity and iterativity.

Let us start with the recursive one. For an expression like *Every boy gave things …*, it seems to be necessary to nest one projection in the other. The following implementation clearly shows that even if the variables are specified correctly, one gets varying results for the direct object (which would also be the case in same-size results).

?- setof(X,{X,DO}/setof(Y,{X,Y}/give_to(E,X,Y,Z),DO),SU).

DO = [[t1, t11]],

SU = [[b1]];

DO = [[t2], [t3]],

SU = [[b2]];

DO = [[t4], [t5]],

SU = [[b3]].

In the iterative version, the subject argument is projected as usual, but the projection for the direct object can be treated *independently*, only that the subject variable has to be considered as a further bound variable:

?- setof(Y,{X,Y}/give_to(E,X,Y,Z),DO).

X = [b1],

DO = [[t1, t11]];

X = [b2],

DO = [[t2], [t3]];

X = [b3],

DO = [[t4], [t5]].

**Observation 6.** Distributivity is based on *selection*, that is, restricting projection of some variable y to the value of some other variable x. It implies that x has been put on a store of bound variables used in the selection.

### 3.4 Multi-collections

Obviously, such a procedure allows direct generation of some DO, but still is a distributed result overall. The solution to this problem is to find all dependent collections and collect them into one, using the built-in findall-predicate^12^:

?-findall(DO,setof(Y,{X,Y}/give_to(E,X,Y,Z),DO),DOs).

DOs = [[[t1, t11]], [[t2], [t3]], [[t4], [t5]]].

(18) shows the predicative part of 〚*give_to*〛 that still is in need for a generalized treatment of the bound variables in projections.

(18) $\begin{array}{c} \begin{array}{l} \text{findall}\left(\text{su},\text{set}\text{of}\left(\text{x,}\left\{\text{x}\right\}\text{/give\_to}\left(\text{e,x,y,z}\right)\text{,su}\right)\text{,SU}\right)\\ \text{findall}\left(\text{do,setof}\left(\text{y,}\left\{\text{x,y}\right\}\text{/give\_to}\left(\text{e,x,y,z}\right)\text{,do}\right)\text{,DO}\right)\\ \text{findall}\left(\text{po,setof}\left(\text{z,}\left\{\text{x,z}\right\}\text{/give\_to}\left(\text{e,x,y,z}\right)\text{,po}\right)\text{,PO}\right) \end{array} \end{array}$

Evidently, the generation perspective has already led us into new ground: aside from event participant instances as collections (not individuals), and collections of collections as basis for quantification, we apparently have to assume so-called *multi-collections* capturing the variance of dependent event participants. With the scenario, the complexity has been before our eyes all along: different from typical simple *Every man loves a woman* examples, it requires far more differentiated quantifiers. But, do we really need to assume multi-collections? Perhaps, it is naïve to assume representational reality for this distribution variation.

Actually, we do. Let us say we want to be more specific about our scenario, distributing over the collection of subject instances, but possibly cumulating over the others. This is less interesting with respect to the direct object, as in (19a), because the type ‘two things’ is the same. With respect to the prepositional object, however, the information about the girls can be expressed more differentiatedly, as in (19b). This shows that there must be a range of degrees, which can only originate from a multi-collection.

(19)
  a. Every boy gave (*on the whole*) *two things* to some girls.
  b. Every boy gave some things to (*on the whole*) ***one to three girls*** (***/at least one girl /at most three girls***).

**Observation 7**. The result of a projection is more complex than a collection of event participant instances. It is a collection of such collections (a *multi-collection*).

### 3.5 Nondistributivity and Noncumulativity: The Case of German *jeweils*

I have deliberately added “(*on the whole*)” to the examples in (19) because although there is distributivity with respect to the subject, the corresponding quantifiers are cumulative here (with accumulation across events). Accordingly, there is an even more specific generalization for the verbalization of the scenario, lacking such cumulativity, as shown in (20).

(20) Die Jungen gaben Sachen an jeweils ein bis zwei Mädchen.
The boys gave things to, in each case, one to two girls.
“The boys gave things to one to two girls each/on each occasion.”

In contrast to (19), (20) conveys the information about the range of the number of girls *per event*, not (only) per boy. Unfortunately, we are entering uncharted territory here, concerning both the unsettled semantics of *jeweils* and the cross-linguistic correspondences involved. In addition to that, different kinds of distributivity (uses of *each*) can easily be confused.

Champollion (to appear) reviewed the use of distributivity markers in different languages and gives translation examples with *jeweils* (his (9) and (10) as (21) and (22), respectively). According to him, the German marker can be translated either as adnominal *each*, as *on each occasion*, or as *each time*.

(21) Die Redakteure haben jeweils sechs Fehler entdeckt.
The copy editors have DIST six mistakes discovered.
  a. “Each of the copy editors caught six mistakes.”
  b. “The copy editors have discovered six mistakes on each salient occasion.”
(22) Der Redakteur hat jeweils sechs Fehler entdeckt.
 The copy editor has DIST six mistakes discovered.
 “The copy editor caught six mistakes each time.”

*jeweils* might also partially correspond to the binominal *each* of Safir and Stowell’s (1988) example in (23) (my gloss/translation). This was proposed by Kobele and Zimmermann (2012), who rule out *each* as a translation of adverbial *jeweils* in (24) [ (196) in their work].

(23) Two men saw two women each.
 Zwei Männer sahen zwei Frauen je.
 “Zwei Männer sahen je*(weils) zwei Frauen.”
(24) Die Jungen haben je*(weils) gewonnen.
 The boys have each won.
 “The boys won each time.”
 (not: “Each boy won.”)

There are some objections to these analyses, however. First, *each time* is not a standard translation of *jeweils*. Instead, German temporal quantifier words mostly include *-mal*: *each/every time* (*jedesmal*), *one/two/…time(s)* (*ein-/zwei-/…mal*), *many times* (*viele Male*), *oftentimes* (*oftmals*), etc. Second, instead of *jeweils*, the correct translation of floating *each* in (21) is *jeder*, which is, conversely, corroborated by (24). Third, and most importantly, it is not the case that *jeweils* is distributive, as is wrongly stated in (21a). Both here, and in (23), distributive *each* would have to be translated by *jeder* (*Die Redakteure haben* jeder *sechs Fehler entdeckt*, *Die Männer sahen* jeder *zwei Frauen*).

Nondistributive *jeweils* (“on each occasion”) therefore has to be distinguished from distributive *je (zwei/drei)* (“each (two/three)”) and *jeder/jede/jedes* (“each”). To show that, one can extend the example with a cumulative PP [see (25)]. Assume that there are reading sessions (some copy editors reading five documents). As (25a) shows, global cumulativity of the documents is not guaranteed.^13^ With *jeweils* in (25b), however, it is: the sentence asserts six mistakes per session but (correctly) leaves the number of editors per session open.

(25)
  a. Die Redakteure haben jeder sechs Fehler in (insgesamt) fünf Dokumenten entdeckt.
  The copy editors have DIST six mistakes in (on the whole) five documents discovered.
  “Each of the copy editors caught six mistakes in five documents.”
             (not necessarily: 5 docs in total)
  b. Die Redakteure haben jeweils sechs Fehler in (insgesamt) fünf Dokumenten entdeckt.
  The copy editors have on each occasion six mistakes in (on the whole) five documents discovered.
  “In five documents overall, the copy editors caught six mistakes on each occasion.”

**Observation 8**. *Each* is not a translation of *jeweils* in most, if not all, relevant structural positions, and neither is *each time*. While *each* is distributive, *jeweils* is not.

Zimmermann (2002) presented an extensive discussion of the semantics of *jeweils* (which he notes is less restricted than *each*). Yet by confounding *jeweils* (*on each occasion*), *jeder (je zwei/…) (each (two/…)*), and *jedesmal* (*each time*), his analyses do not lead to the conclusion I would like to offer in the following. The distinct contribution of *jeweils*, as opposed to *each*, can best be demonstrated with examples like those in (26).

(26)
  a. Je zwei Personen deckten (insgesamt) zwölf Festessenstische.
  Each two persons set the table for (on the whole) twelve             banquet tables.
  (=|PERSONS|/2×12 tables).
  b. Jeweils zwei Personen deckten insgesamt zwölf Festessenstische.
  On each occasion, two persons set the table for, on the            whole, twelve banquet tables.
  (=12 tables).
  c. Sechs Personen deckten (jeweils) zu zweit insgesamt zwölf Festessenstische.
  Six persons set the table pairwise for, on the whole, twelve            banquet tables.
  (=12 tables).

(26a) distributes over the persons. So, if there are six of them, there must be thirty-six tables, despite the verbalized total of twelve (which can be called *local cumulativity*, dependent on some pair of persons). According to (26b), there may also be six persons, but the number of tables will always be twelve. Yet, it asserts that, in each table setting, two persons are involved. Finally, post-nominal qualifiers such as *pairwise* and *individually*, as in (26c), can be analyzed as elements expressing the size of the event participant instances, to be distinguished from distributive elements such as *each* (which would imply a larger number of tables).

These examples show that there is a characteristic distinction between *jeweils* and *jeder/je X* in that only the latter is distributive. Therefore, it is *not* the case that “the presence of *jeweils* disambiguates, in favor of distributivity, the interpretation of sentences which otherwise would be ambiguous between a distributive interpretation and a collective one” (Kobele and Zimmermann 2012, p. 260). Rather and in contrast to *insgesamt* (*on the whole*), it disambiguates quantified NPs as being noncumulative, rather than being cumulative. It can be confused with *each* because both are noncumulative, but only the latter is distributive.

**Observation 9**. *jeweils* marks noncumulativity (but not distributivity), *insgesamt* (*on the whole*) cumulativity.

### 3.6 Quantification Levels

*jeweils* somehow puts a focus on an event participant by measuring the size of the instance(s), allowing cumulativity with respect to the other event participants [see (27a-c), with (27) describing the same scenario].^14^ As (27d) shows, distributivity with respect to the subject may lead to *local* cumulativity of the other event participants. Therefore, this can best be depicted as describing the *same* situation by expressing quantification information *on different levels* (global cumulative vs. local cumulative vs. (distributive) event level) determined by the selection restrictions on projection.^15^

(27)
  a. Jeweils zwei Helfer gaben hunderte Carepakete an tausende Flüchtlinge aus.
  On each occasion two helpers gave hundreds of Care packets to thousands of refugees out.
  b. Dutzende Helfer gaben jeweils ein bis zwei Carepakete an tausende Flüchtlinge aus.Dozens of helpers gave on each occasion one to two Care packets to thousands of refugees out.
  c. Dutzende Helfer gaben hunderte Carepakete an jeweils drei bis vier Flüchtlinge aus.
  Dozens of helpers gave hundreds of Care packets to on each occasion three to four refugees out.
  d. Je zwei Helfer gaben dutzende Carepakete an hunderte Flüchtlinge aus.Each two helpers gave dozens of Care packets to hundreds of refugees out.

Procedurally, event-level quantification corresponds to projecting an event participant variable with the referential event variable being bound. Observe that, in the following <A>, this leads to small multi-collections, which slightly differ from the local cumulativity in distributive <B> (where the presents of b2 and b3 are grouped, respectively). <C> contains a single multi-collection, the global cumulus of present collections.

<A>

?-findall(DO,setof(Y,{E,Y}/give_to(E,X,Y,Z),DO),DOs).

DOs = [[[t1, t11]], [[t2]], [[t3]], [[t4]], [[t5]]].

<B>

?-findall(DO,setof(Y,{X,Y}/give_to(E,X,Y,Z),DO),DOs).

DOs = [[[t1, t11]], [[t2], [t3]], [[t4], [t5]]].

<C>

?-findall(DO,setof(Y,{Y}/give_to(E,X,Y,Z),DO),DOs).

DOs = [[[t1, t11], [t2], [t3], [t4], [t5]]].

**Observation 10.** Apart from, and sometimes in addition to, measurement aspects of collections, quantifiers allow to transport information about a projection on different levels of granularity [global cumulative vs. local cumulative vs. (distributive) event level], to adapt to the variation of different scenarios and foci of interest. Local cumulativity is cumulativity in distributive scope.

### 3.7 Generating Quantifiers

For a demonstration of the impact of taking a generation perspective on quantification and on the interplay of its aspects, I have implemented a procedure describeScenario that simply iterates through all possibilities of projection with or without selection options and directly generates quantified sentences. There are some provisos, however.

First, simply for the sake of readability, the output consists of direct translations of acceptable German sentences instead of German sentences glossed in English. It also includes explicit markers of quantification options to avoid ambiguities (*on the whole*), even if they would probably be omitted in natural sentences for pragmatic reasons. Both aspects facilitate recognizing similarities and differences in each case. Second, I did not even try to give acceptable English translations due to the known cross-linguistic differences (which would require perfect competence of English and furthermore would rather distract from the point under discussion). Third, I did not use a grammar for generation because that would presuppose the solution of some of the structural puzzles still under investigation (see Zimmermann, 2002 for the case of *each* and *jeweils* in generative linguistics).

Fourth, I restricted the set of quantification expressions to consider for generation. Expressions like *all/each/every …* are not included because they are presuppositional (#*All boys gave all presents to all girls*). Although this could have been easily amended by setting some context (*There are three boys…*), this would be relevant only for the givers and is, therefore, not that interesting overall. Expressions like *most/many/few …* are excluded for similar reasons: they presuppose class- and situation-related knowledge about typical collection sizes, other degrees on the scale, etc. I also left out default indicators like *a few* and bare plurals (*Boys gave things to girls*). Finally, singular descriptions do not appear at all because of the summary descriptions always leading to set sizes greater than one.^16^ This is remarkable because such descriptions belong to the prominent type of existential quantification. With these provisos, here are the descriptions automatically generated for the above scenario:

?- describeScenario.

Possible Descriptions:

d1: some boy(s) gave some thing(s) to some girl(s)

d2: some boy(s) gave some thing(s) to on the whole 4 girl(s)

d3: some boy(s) gave some thing(s) to on each occasion 1 to 2 girl(s)

d4: some boy(s) gave on the whole 6 thing(s) to some girl(s)

d5: some boy(s) gave on the whole 6 thing(s) to on the whole 4 girl(s)

d6: some boy(s) gave on the whole 6 thing(s) to on each occasion 1 to 2 girl(s)

d7: some boy(s) gave on each occasion 1 to 2 thing(s) to some girl(s)

d8: some boy(s) gave on each occasion 1 to 2 thing(s) to on the whole 4 girl(s)

d9: some boy(s) gave on each occasion 1 to 2 thing(s) to on each occasion 1 to 2 girl(s)

d10: some boy(s) each gave some thing(s) to some girl(s)

d11: some boy(s) each gave some thing(s) to on the whole 1 to 3 girl(s)

d12: some boy(s) each gave some thing(s) to on each occasion 1 to 2 girl(s)

d13: some boy(s) each gave on the whole 2 thing(s) to some girl(s)

d14: some boy(s) each gave on the whole 2 thing(s) to on the whole 1 to 3 girl(s)

d15: some boy(s) each gave on the whole 2 thing(s) to on each occasion 1 to 2 girl(s)

d16: some boy(s) each gave on each occasion 1 to 2 thing(s) to some girl(s)

d17: some boy(s) each gave on each occasion 1 to 2 thing(s) to on the whole 1 to 3 girl(s)

d18: some boy(s) each gave on each occasion 1 to 2 thing(s) to on each occasion 1 to 2 girl(s)

d19: some boy(s) individually gave some thing(s) to some girl(s)

d20: some boy(s) individually gave some thing(s) to on the whole 4 girl(s)

d21: some boy(s) individually gave some thing(s) to on each occasion 1 to 2 girl(s)

d22: some boy(s) individually gave on the whole 6 thing(s) to some girl(s)

d23: some boy(s) individually gave on the whole 6 thing(s) to on the whole 4 girl(s)

d24: some boy(s) individually gave on the whole 6 thing(s) to on each occasion 1 to 2 girl(s)

d25: some boy(s) individually gave on each occasion 1 to 2 thing(s) to some girl(s)

d26: some boy(s) individually gave on each occasion 1 to 2 thing(s) to on the whole 4 girl(s)

d27: some boy(s) individually gave on each occasion 1 to 2 thing(s) to on each occasion 1 to 2 girl(s)

d28: on the whole 3 boy(s) gave some thing(s) to some girl(s)

d29: on the whole 3 boy(s) gave some thing(s) to on the whole 4 girl(s)

d30: on the whole 3 boy(s) gave some thing(s) to on each occasion 1 to 2 girl(s)

d31: on the whole 3 boy(s) gave on the whole 6 thing(s) to some girl(s)

d32: on the whole 3 boy(s) gave on the whole 6 thing(s) to on the whole 4 girl(s)

d33: on the whole 3 boy(s) gave on the whole 6 thing(s) to on each occasion 1 to 2 girl(s)

d34: on the whole 3 boy(s) gave on each occasion 1 to 2 thing(s) to some girl(s)

d35: on the whole 3 boy(s) gave on each occasion 1 to 2 thing(s) to on the whole 4 girl(s)

d36: on the whole 3 boy(s) gave on each occasion 1 to 2 thing(s) to on each occasion 1 to 2 girl(s)

d37: on the whole 3 boy(s) each gave some thing(s) to some girl(s)

d38: on the whole 3 boy(s) each gave some thing(s) to on the whole 1 to 3 girl(s)

d39: on the whole 3 boy(s) each gave some thing(s) to on each occasion 1 to 2 girl(s)

d40: on the whole 3 boy(s) each gave on the whole 2 thing(s) to some girl(s)

d41: on the whole 3 boy(s) each gave on the whole 2 thing(s) to on the whole 1 to 3 girl(s)

d42: on the whole 3 boy(s) each gave on the whole 2 thing(s) to on each occasion 1 to 2 girl(s)

d43: on the whole 3 boy(s) each gave on each occasion 1 to 2 thing(s) to some girl(s)

d44: on the whole 3 boy(s) each gave on each occasion 1 to 2 thing(s) to on the whole 1 to 3 girl(s)

d45: on the whole 3 boy(s) each gave on each occasion 1 to 2 thing(s) to on each occasion 1 to 2 girl(s)

d46: on the whole 3 boy(s) individually gave some thing(s) to some girl(s)

d47: on the whole 3 boy(s) individually gave some thing(s) to on the whole 4 girl(s)

d48: on the whole 3 boy(s) individually gave some thing(s) to on each occasion 1 to 2 girl(s)

d49: on the whole 3 boy(s) individually gave on the whole 6 thing(s) to some girl(s)

d50: on the whole 3 boy(s) individually gave on the whole 6 thing(s) to on the whole 4 girl(s)

d51: on the whole 3 boy(s) individually gave on the whole 6 thing(s) to on each occasion 1 to 2 girl(s)

d52: on the whole 3 boy(s) individually gave on each occasion 1 to 2 thing(s) to some girl(s)

d53: on the whole 3 boy(s) individually gave on each occasion 1 to 2 thing(s) to on the whole 4 girl(s)

d54: on the whole 3 boy(s) individually gave on each occasion 1 to 2 thing(s) to on each occasion 1 to 2 girl(s)

d55: on each occasion 1 boy(s) gave some thing(s) to some girl(s)

d56: on each occasion 1 boy(s) gave some thing(s) to on the whole 4 girl(s)

d57: on each occasion 1 boy(s) gave some thing(s) to on each occasion 1 to 2 girl(s)

d58: on each occasion 1 boy(s) gave on the whole 6 thing(s) to some girl(s)

d59: on each occasion 1 boy(s) gave on the whole 6 thing(s) to on the whole 4 girl(s)

d60: on each occasion 1 boy(s) gave on the whole 6 thing(s) to on each occasion 1 to 2 girl(s)

d61: on each occasion 1 boy(s) gave on each occasion 1 to 2 thing(s) to some girl(s)

d62: on each occasion 1 boy(s) gave on each occasion 1 to 2 thing(s) to on the whole 4 girl(s)

d63: on each occasion 1 boy(s) gave on each occasion 1 to 2 thing(s) to on each occasion 1 to 2 girl(s)

There are two reasons for the existence of this subsection and, especially, this listing. First, it is supposed to be a demonstration *ad oculos* of the generationist scheme of quantification, exemplifying the interplay of collectivity, global cumulativity (*4 girl(s)*), distributivity with local cumulativity (*1 to 3 girl(s)*), and event-level noncumulative quantification (*1 to 2 girl(s)*) with multi-collections; this includes the markers of distributivity (*each*), nondistributivity (*individually*), cumulativity (*on the whole*), and noncumulativity (*on each occasion*). Note that multi-collections are verbalized both in local cumulativity (*on the whole, 1 to 3 girl(s)*) and event-level noncumulative (*on each occasion, 1 to 2 girl(s)*) settings reflecting the corresponding variance.

Accordingly, it is not intended to showcase a certain approach of a method or implementation handling quantification, or even a certain new natural language generation approach of generating English quantified sentences. I am, of course, open to any quite different (probably more effective) method, or, in times of Deep Learning, to any other type of implementation.

Linguistically, as said in the provisos, it is a crutch (see also the technical preliminaries of Section 3). Yet, while the “English sentences” are bad English, their German translations would be nearly perfect. Note, however, that the German equivalent of “individually” is ill-placed at that position in a German sentence (it cannot occur post-nominally, but rather appears in “floating” positions). Hence the simplified listing with all red flags set to prevent such discussions.^17^

Second, the implementation presented here must not be taken as the goal or result of the article. It should rather be viewed as a method on the theory/knowledge level in the sense of “prototyping as theory building”. Starting with the idea to apply the generation view to the field of quantification, this provided the means to test, monitor, and refine the generation view straightforwardly. I regard this as eminently effective methodologically (see also Lang et al., 1991; Carstensen, 2001) and can definitely recommend it, especially in the field of language and computation with its vast amount of related approaches on different levels and in different disciplines (linguistics, computational linguistics, AI, logic, and computer science).

## 4 Discussion

### 4.1 General Aspects

Let me summarize the main points made in the previous section. Quantification can basically be regarded as measuring the collection of instances of some framed event’s participant variable. Collections as such exist on three levels (instance, collection, and multi-collection). While *collectivity* is a phenomenon on the instance level, *cumulativity* concerns the (multi-)collection level. The procedural options of implementation showed that cumulativity—understood as a basic phenomenon of collecting instances for a summary description (and, therefore, rather a default phenomenon)—can be regarded as resulting from a *projection* of a predicate’s relation framing the scenario, chosen by the speaker. Adding *selection* to the projection may lead to *distributivity*, which, besides setting the event level, involves keeping track of the corresponding event participant variable as a bound variable in subsequent projections (showing *local* cumulativity) of an ordered list of such variables. Temporary selection of the referential event argument variable sets event level, corresponding to a local perspective on the scene, allowing for a *noncumulative* specification of the common size of the instances (e.g., *on each occasion two/…*) and for *global* cumulativity in further projections.

This scheme departs in various respects from the FOPL/GQT tradition. It clearly separates variable-binding and quantification proper, and assigns variable-binding a more technical role. It also disassociates *existence* from both of these concepts and leaves it open to (philosophical) discussion whether existence should still be treated as variable-binding.

The central idea of the generation view can already be found in the practical/computational (linguistics) approach of Scha and Stallard: “Noun phrases, regardless of number, quantify over sets of individuals […] Verbs can now be uniformly typed to accept sets of individuals as their arguments” (Scha and Stallard, 1988, p. 18). This greatly simplifies (“flattens”) the *compositionality* of verbs and noun phrases, and keeps lexical-level arguments (collection type) and concept-level arguments (instance collection type) apart. It also obviates the need for the full power of the lambda calculus.

Along with the “quantification as measurement” view, this compositional treatment allows a semantic analysis of quantifier expressions paralleling those of gradation expressions (see (2)). As a corollary of that, the determiner/modifier debate about the syntactic function of quantifier expressions (Krifka, 1999) is rendered obsolete. Not only do their parts of speech vary anyway but also their possible *complexity* (*almost every, many more than twenty,* etc.) has been underrated/neglected for the most part. Besides that, quantification information can evidently be distributed on different forms (*three boys individually*) in various positions (e.g., *each*) in a nonuniform way (almost all/every vs. *almost each).

Unlike the GQT conception of quantifiers as relating properties (involving set intersection), quantification is seen as characterizing a complete conceptual cover of a projection. Projections presuppose a relation of ordered event participants corresponding to a framed scenario/situation as verb (sense) denotation. The projections are kin both to the summation operator in (6) and the generalized distribution operator in (9). Both are critically discussed in the literature, however (for an overview, see Champollion, 2019).

As to summation, the generation view shows that one needs an actual, parametrizable operation of *collection*, in addition to just assuming (elements of) a complex domain. The compositional, partial *distribution* operator has turned out to be not only too general but also superfluous in the proposed scheme (as the examples can/must be analyzed as cases of cumulativity). This is evident in (28), which is the slightly extended equivalent description for the above example in (10).

(28) Diese insgesamt sechs Eier kosten jeweils 2 €.
These, on the whole, six eggs cost, in each case, € 2.
“These six eggs cost € 2 (‘distributive’ reading).”

Instead of distributivity, cumulativity and event-level selection are used (and linguistically marked) to indicate the same costs of different egg collections. Note that this includes *collectivity* as a necessary ontological aspect.

The realistic scenario used in the previous section immediately demonstrated the impact of respecting the variance in the event participant instances, and its description clarified the necessity to assume a further level of *multi-collections* and their expression (especially in the case of distributivity). Accordingly, quantification can also be regarded as operating on different levels (the so-called *quantification levels*), by using projection and selection selectively to adapt to, and focus on, relevant aspects of the scenario.

According to the generation view, projections *construct* the NP denotations, and the fixed order of the event participants can be regarded as the source of scope asymmetry effects. *Scope*, in general, is disentangled from the (linear) order of (variable-binding) quantifiers. With nondistributive event-level quantification, a corresponding solution to the problem of partially ordered (branching) quantifiers is offered. (29) shows a (perfect) corresponding German verbalization (cumulative markers omitted) of the examples in (12). Although there is still some indeterminacy/underspecification of the actual scenario relation, there is no forced over-distributivity anymore.

(29) Jeweils einer von drei Jungen und jeweils eines von vier Mädchen hassen sich.
In each case, one of three boys and, in each case, one of four       girls hate each other.
“Three boys and four girls hate each other (intended        meaning).”

Crucial to this treatment is the idea to view both event-level quantification (*in each case*) and distributivity as involving a bound event variable in the projection (distributivity adding keeping track of it). Unlike the sense of ‘distributing application of a property to (atomic) elements of a cover’ interfering with semantic composition, distributivity is, therefore, seen here as a more basic result of parametrizing projections to treat argument variables/positions as bound. It is one of the main results of this investigation that the ‘distribution sense’ is insufficient to account for the range descriptions despite (described parts being in) distributive scope. These descriptions rather imply the existence of the so-called *multi-collections* that go across distributed predications. To regard distribution as a parameter/feature of the collection operation (rather than as a distributing operator) is a unique aspect of this scheme, which might be independently motivated by the variety of distributive marker positions shown in (8).

Davidsonian events play an important role in projection-based quantification, allowing for event-level representations and descriptions. The referential event arguments are different from event participant arguments, however; as there are no plural event expressions (*Peter three/many/… jumped), the existence (and quantification) of event pluralities as “verbal pluralities” is denied here. Instead of that, event pluralities are assumed to appear only as accumulations of event participants, including space/time/plexity roles (“three *place,*” “often *times,*” and “many *fold*”). Or they appear as “objectivized” events in the nominal domain (*Peter’s three/frequent/many jumps*) (see also Carstensen, 2011). While only basic events are considered here (note that I generally left out the verb’s event variable), others, like Tunstall (1998); Kratzer (2007), emphasize the relevance of complex event structures.

Working systematically with a realistic scenario showing some variance had the side effect of discovering not only multi-collections but also the role of the nondistributive *jeweils* (*on each occasion*) setting ‘event level’ for finer-grained descriptions and of *insgesamt* (*on the whole*) signaling non-event-level (cumulativity). Likewise, expressions like *individually* and *in pairs* were found to characterize the collection element size nondistributively on the event level.

### 4.2 Cognitive Aspects

As a cognitivist position, the present approach is different from theories that simply map language to the world truth- or model-theoretically. It assumes primacy of speaking/generation over interpretation, processes that operate on explicit representations of the world, and an indirect access to the latter (Lang and Maienborn, 2011). It also takes quantifiers to be far more complex and heterogeneous than, most of all, GQT (see also Feiman and Snedeker, 2016).

For example, while *all* and *every* are typically treated as determiners, *almost all* and *almost every* show that they rather denote the maximal degree of the quantity scale than a relation between properties. This is why quantification should better be modeled as analogous to gradation in the adjectival domain [see (15) and (16), and Carstensen (2013)]. According to that, *all* and *every* both explicitly refer to the class-related scale of the collection. *all* allows both global and local perspectives (defaulting to the former), while *every* sets local perspective and distribution. *Each* is likewise distributive, but focuses on the atomic event participants, disregarding gradation aspects of the class-related scale (*almost each). This is different again with *individually*, which is semantically rather a condition of collections to consist of singletons only (local, nondistributive), and with *together*, which requires instance size to be equal to collection size.

It is less surprising, therefore, that singular quantifiers can be used for a factual plurality.^18^ Rather than expressing a distinction between individuals and pluralities, singular and plural indicate different perspectives (here, on the instance level). In line with proposals made by others (discussed in Nouwen, 2014), plural can be seen as making no restriction on the size of the event participant instances, while singular requires an instance to be atomic (of size one). This is a perspective/constraint, however, because the overall collection size can be zero [in which case both perspectives are possible, see (30)].

(30) There {is/are} (almost) no {cloud/clouds} in the sky.

According to the cognitivist position, one not only has to distinguish world-, conceptual representation-, and linguistic level, there are also complex mappings between world and representation, and representation and language, respectively. For example, the same situation can be categorized as being about pairs of objects (as *groups*) or about collections of two objects resulting in different expressions (*pairs of …* vs. *each two …* ). With respect to the count/mass distinction, Pelletier argued that “philosophical and linguistic semanticists would like to have some input from psychological studies” (see Pelletier, 2010, p. 168). Starting out as a quest for corresponding ontological distinctions, Carstensen (2011) ended with the result that they must be conceived as relative to attentional perspectivation. It was also found that ‘object’ and ‘singular’, and ‘collection’ and ‘plural’, respectively, are both related, but nonidentical notions, since the binary linguistic distinction (singular/plural) does not match the quaternary top ontological distinction (object/group/collection/stuff).^19^ This mismatch can be pinpointed as the reason for cross-linguistic differences in the transition area between singular and plural, observable, for example, in the existence of *dual* morphology to mark two elements in some languages, or in cross-linguistic lexical divergences in grammatical number [English *scissors, trousers* (pl.) vs. German *Schere, Hose* (sg)].

As has been shown with *each* and *every*, the mapping to language is complex in quantification, too. Different syntactic positions (e.g., of *each*) and different parts of speech (compare almost no thing/almost nothing/*almost not a thing) allow to transport differential aspects of the content, given some aspects of the world, some constraints of the linguistic context, or some needs of the hearer. This is quite different from wholistic conceptions of quantifiers, either FOPL’s individual-variable-binding operators or the generalized quantifiers of GQT.

### 4.3 Semantic Aspects

Despite the fact that the PROLOG code can be read declaratively, the present approach is clearly procedural due to the notion of ordered projections of verb arguments. However, each result, such as a collection of instances covered by a common class concept, is quite comparable to the declarative notion of a sum of individuals being in the denotation of a starred nominal predicate, and so is the projection-based linkage of argument collections and their frame predicate to generalized verbal predicates. Both perspectives therefore somehow meet in the preliminary semantic representation (31) of the sentence *Three boys each gave things to girls.*

(31) $\begin{array}{c} \begin{array}{l} \text{Pred}=\text{give\_to}\left(\text{e},\text{x},\text{y},\text{z}\right)\\ \text{BV}=Ø\\ \text{collection}\left(\text{x},\left\{\text{x},\text{e}\right\}⋃\text{BV}/\text{Pred},\text{SU}\right)\\ {}*\text{boy}\left(\text{SU}\right)\&\text{meas}\left(\text{SU}\right)=\text{d}\&\text{d}\approx d3\\ \text{Atoms}\left(\text{SU},1\right)\&\text{Distr}\left(\text{x},\text{BV}\right)\\ \text{collection}\left(\text{y},\left\{\text{y}\right\}⋃\text{BV}/\text{Pred},\text{DO}\right)\&\text{*thing}\left(\text{DO}\right)\\ \text{collection}\left(\text{z},\left\{\text{z}\right\}⋃\text{BV}/\text{Pred},\text{PO}\right)\&\text{*girl}\left(\text{PO}\right)\\ {}**\text{give\_to}\left(\text{SU},\text{DO},\text{PO}\right) \end{array} \end{array}$

In (31), the procedural details of collecting instances are hidden in a declarative “collection” predicate. “BV” is the store of bound variables, initialized as empty. “Distr” is an operator putting a variable on the store. In the subject collection, “e” is temporarily bound, setting event level. Tentatively, “Atoms (C,N)” characterizes a collection C as consisting of elements of size N.

In the present proposal, therefore, standard distributivity consists of three conditions: setting event level, putting a variable on store “BV,” and specifying the common size of the instances of the collection (here, 1 for *each*). “Atoms (C,N)” could then be defined as not(x∈C&not(|x|=N)).

Unfortunately, this semantic representation is defective in various respects. For example, it is unclear how *multi-collections* fit in the picture. In describeScenario, the collections (in a multi-collection) are simply treated by measuring them, building an ordered set of measures, and verbalizing the corresponding range with a path description (an abstract directional, see Carstensen, 2019). The difference of collection and multi-collection is disregarded in (31) and, generally, in need of analysis and formal explication.

While the first line of (31) is comprehensible as an abbreviation, it is not interpretable at all. This points to the fact that the whole idea of pre-semantic accessing the frame concept and specifying some of its variables for projection/selection is formally unclear, especially in semantic composition. Also, the last line is basically superfluous because the relationship of the collections to the frame predicate (or *R*) is given in the collection predicates. Finally, the order of the projections is not fully reflected/guaranteed in the declarative (31).

And yet, there should be ways to amend the addressed points. For example, the variables of the frame predicate could simply be hidden on the linguistic level, and information about distribution and event selection could be represented and relayed by features/indices [as indicated in (32)]. Projections could be specified by argument numbers of the concept (or, probably more appropriate anyway, *via* thematic roles; see Parsons, 1995 for such a Neo-Davidsonian approach). Then, if realization of different syntactic functions is ensured, the semantics of a specific syntactic form of *give* could be represented as in (32), which ultimately boils down to Link/Krifka approaches like (33) to be defined accordingly. Thus the real—and hard—work probably lies in adapting quantifier logics to this new view of quantification.

(32) $〚give\_to〛=\;\mathrm{\lambda P}O^{pd,pe}\lambda DO^{dd,de}\lambda {\text{SU}}^{sd,se}\begin{array}{c} \begin{array}{l} \text{collection}\left(\text{give\_to},1,{\text{SU}}^{sd,se}\right)\\ \text{collection}{(\text{give\_to},2,\text{DO}}^{dd,de})\\ \text{collection}{(\text{give\_to},3,\text{PO}}^{pd,pe}) \end{array} \end{array}$
(33) $〚give\_to〛=\mathrm{\lambda P}O^{pd,pe}\lambda DO^{dd,de}\lambda SU^{sd,se}\begin{array}{c} \begin{array}{l} {}**\text{give\_to}\left({\text{SU}}^{sd,se},DO^{dd,de},PO^{pd,pe}\right) \end{array} \end{array}$

The present investigation has been deliberatively restricted (see Section 3.7), assumedly without loss of generality. For example, spatiotemporal (*everywhere, three times*) and other aspects of basic events are left out, as are event aspects of the summed verbal predicate (*Yesterday/In the kindergarten in Maine Street…*, see Kratzer, 2007 for a discussion of event analyses with basic events and further event structure). This also holds for aspects of scope (inversion), which is a favorite topic in the interpretative perspective research but often leads to overly general approaches (Steedman, 2012).

Finally, it is a side effect of choosing a realistic scenario that singular indefinite NPs do not appear, as there is no corresponding common type in noncumulative descriptions. Else, descriptions such as *a/the thing* would appear under the premise that there are only atomic collection elements, that measurement is not expressed (*one thing*), and that the language’s grammar excludes singular NPs without a determiner (as is the case in German and English).

## 5 Conclusion

The work documented in this article started with the hypothesis that it is beneficial and even necessary to apply the generation view to the field of quantifiers and quantification in natural language semantics. In a review of this field, severe problems in the interplay of collectivity, distributivity, cumulativity, and plurality in the semantics of quantification expressions were shown, corroborating the hypothesis^20^. For the application of the generation view, the necessary steps toward generating quantification expressions were explicated, and important observations were gathered which collectively characterize the *scheme* of generationist quantification. This scheme was tested with a simple PROLOG prototype for a small, but realistic scenario, resulting in a listing of the *range of verbalization possibilities* according to the scheme and its parameters. After the proof-of-concept demonstration, aspects of the scheme and its implications for the solution of the reviewed problems were discussed.

Some of the ideas presented here are in agreement with many of the current proposals, for example, the uniform treatment of (plural) NPs as involving “plural entities” (i.e., collections), collection-based semantic composition (with projections), the disagreement with some of GQT’s assumptions, and the importance of considering events. It turned out, however, that the generation view highlights or uncovers important aspects of quantification (often) neglected in the interpretative view. Among these are the *role of events and instance collections*, when starting with a nontrivial scenario; the constructive aspects of quantification related to its function as a *summary description* (*projections and selections* on the represented framed scenario to build the *collections* of some event participant variable’s instances); the possibility of a unified view of quantification proper as *measurement of collections*; different *levels* of collections (instance collection, collection, and multi-collection) and of quantification (cumulativity, local cumulativity, and event level); the default character of cumulativity; event-level aspects of distributivity; nondistributive, noncumulative event-level quantification; the role of multi-collections for the description of different-size collections; the role of linguistic markers signaling the corresponding level (*on the whole, in each case*), or the (homogeneous) size of instance collections (*individually, together*). Together with ideas developed independently (ontological aspects, parallelity of quantification and gradation), this scheme presents a unique new view on quantification, and a different stance on the interplay of collectivity, distributivity, cumulativity, and plurality in the semantics of quantification expressions.

Such a view of quantification indicates a need to rethink basic aspects of quantifier logics and semantics in the 21st century, and to redesign them accordingly. It also shows that even a small-scale investigation can have an impact on the domain of language and computation, if it is based on a change of the perspective on the problem(s) that is motivated interdisciplinarily.

## Data Availability

The raw data supporting the conclusion of this article will be made available by the author, without undue reservation.

## Footnotes

1 There is a vast amount of relevant literature here. In general, see, for example, Carstensen et al. (2010); Haaparanta (2011); for a concise overview of computational semantics, see Blackburn and Bos (2005); Bos (2011).

2 An interesting aspect of Link’s proposal is the generalization over objects and stuff. It remains to be seen, however, whether all aspects of the object–stuff difference are captured by it. Note, for example, that I use “collection” as a term for unbounded pluralities and “group” for bounded ones (see Carstensen (2011) for the importance of *boundedness* in semantics and ontology).

3 A prominent example being Krifka’s **-operator for the pluralization of binary predicates, see Beck and Sauerland (2000).

4 That is, one boy might eat his pizzas all at once (say, as a stack), another might eat each one with a pause in between, etc.

5 Correspondingly, these methods are only used as a tool for thinking, that is, theory building and refining (see also Carstensen (1991); Lang et al. (1991); Carstensen (1992); Carstensen, 2000; and Carstensen, 2001).

6 Actually, there is an important built-in predicate setof in PROLOG; with setof (X,prop('class','boy(s)',X),S), one can get the corresponding set of boys in the actual knowledge base as a list. Accordingly, S will be unified with [b1,b2,b3].

7 A (bounded) group must be ontologically distinguished from a (unbounded) collection [only the former is object-like, compare *the team, *many team, many team members*, cf. Carstensen (2011)]. Hence, the list [t1,t11] cannot represent a group, which rather would have to be represented as [gr1] or so. Cognitively, it corresponds to the set of referents attended to at the event’s space-time (time and space aspects left out here).

8 Note that we are not interested in the verbalization of the single events (actually, this would be quite boring). Finding a *pattern* in the events—the common action and framing perspective—is the prerequisite for nonboring descriptions.

9 See Takac and Knott (2016), for a modern (and more complex) approach to sentence generation of events involving ordered attention to individuals leading to structured representations involving semantic roles.

10 As explained there, the empty head is analyzed as a nonovert indistinguishability relation that neither corresponds to equality nor to standard “at least” analyses. Instead, it allows for finer-granular corrections in both directions (*…three dozens of palettes…. Well, 35/37 palettes, to be exact*).

11 This is of course a simplified view of generalization that would fail to classify the things given in the scenario functionally as “presents.” Accordingly, there are no “presents” in the scenario (descriptions).

12
findall exhaustively applies its second argument (here, the setof-predicate) and collects all instances of its first argument (here, DO) into the list in its third argument (here, DOs) representing the *bag* of solutions.

13 Compare *#Every man loves, on the whole, 25 women* for the case of 25 men each loving a single woman, even if the women are different.

14 The cumulative phrases could be marked with *insgesamt/on the whole*, which is left out here.

15 This is not identical to the multiple levels of plurality in Scha and Stallard (1988).

16 Note also the simplification/impreciseness concerning the singular–plural distinction. One probably has to distinguish *some girl* and *some girls*, and to rule out such descriptions in distributive contexts such as d10: although each boy gives more than one thing, there is only one girl for b1.

17 Actually, a reviewer at first complained about “errors” in the listing of these “English sentences”.

18 As in *each* and *every*, but also in *many a* [corresponding to the German singular expressions *jeder* and *mancher* (which also has a plural form)].

19 Example terms for ‘group’ are *team and family*. ‘Collection’ corresponds to plurals or plural nouns such as *cattle*.

20 This is lax for saying that the null hypothesis “no problems in interpretative-view quantification” was refuted.

## References

[B1] Beck S. Sauerland U. (2000). Cumulativity is needed: a reply to winter. Nat. Lang. Semantics 8, 349–371. 10.1023/a:1011240827230

[B2] Blackburn P. Bos J. (2005). Representation and Inference for Natural Language. A First Course in Computational Semantics (Stanford, CA: CSLI),

[B3] Boolos G. (1984). To be is to be a value of a variable (or to be some values of some variables). J. Philos. 81, 430–449. 10.2307/2026308

[B4] Bos J. (2011). A survey of computational semantics: representation, inference and knowledge in wide-coverage text understanding. Lang. Linguistics Compass 5, 336–366. 10.1111/j.1749-818x.2011.00284.x

[B7] Carstensen K.-U. (1991). Aspekte der Generierung von Wegbeschreibungen [Aspects of the generation of route descriptions], LILOG-Report 190. IBM Deutschland GmbH.

[B10] Carstensen K.-U. (1992). “Finding adequate routes for the generation of route descriptions,” in KONVENS 92 (1. Konferenz 'Verarbeitung natürlicher Sprache’ [1. conference on ’processing natural language’]), Nürnberg, Germany, October 7–9, 1992. Editor Görz G., 309–318.

[B9] Carstensen K.-U. (2000). “Computing discourse relations on a linguistic basis,” in Presuppositions and underspecification in the computation of temporal and other relations in discourse SFB 340, Report 164. Editor Reyle U., 149–200.

[B11] Carstensen K.-U. (2001). Sprache, Raum und Aufmerksamkeit [Language, Space, and Attention]. Tübingen: Niemeyer Verlag.

[B12] K.-U. Carstensen, Ebert C. Endriss C. Jekat S. Klabunde R. Langer H. (Editors) (2010). Computerlinguistik und Sprachtechnologie: eine Einführung [computational linguistics and language technology: an introduction]. 3rd Edn (Germany: Spektrum Akademischer Verlag).

[B6] Carstensen K.-U. (2011). Toward cognitivist ontologies. Cogn. Process. 12, 379–393. 10.1007/s10339-011-0405-0 21523446

[B8] Carstensen K.-U. (2013). A cognitivist semantics of gradation. Z. für Sprachwissenschaft 32, 181–219. 10.1515/zfs-2013-0007

[B5] Carstensen K.-U. (2019). From motion perception to bob dylan. a cognitivist attentional semantics of directionals. Lo 95, 17–50. 10.13092/lo.95.5514

[B13] Champollion L. (2019). Distributivity in formal semantics. Annu. Rev. Linguist. 5, 289–308. 10.1146/annurev-linguistics-011718-012528

[B14] Champollion L. (to appear). “Distributivity, collectivity and cumulativity,” in The wiley blackwell companion to semantics. Editors Gutzmann D. Matthewson L. Meier C. Rullmann H. Zimmerman T. (The Wiley Blackwell Companions to Linguistics), Vol. 5, 1–38. 10.1002/9781118788516.sem021

[B15] Clocksin W. Mellish C. (1981). Programming in prolog. Berlin, Heidelberg, NY: Springer-Verlag.

[B16] Davidson D. H. (1967). “The logical form of action sentences,” in The logic of decision and action. Editor Rescher N. (Pittsburgh: University of Pittsburgh Press), 81–120.

[B17] Feiman R. Snedeker J. (2016). The logic in language: how all quantifiers are alike, but each quantifier is different. Cogn. Psychol. 87, 29–52. 10.1016/j.cogpsych.2016.04.002 27214380

[B18] Fillmore C. (1977). “Scenes-and-frames semantics,” in Linguistic structure processing. Editor Zampolli A. (Amsterdam: North Holland Publishing Compan), 55–82.

[B19] Haaparanta L. (2011). The development of modern logic. Oxford: Oxford University Press.

[B20] Heim I. Kratzer A. (1998). *Semantics in generative grammar*. Blackwell textbooks in linguistics (United Kingdom: John Wiley & Sons).

[B21] Kamp H. Reyle U. (1993). *From discourse to logic: introduction to modeltheoretic semantics of natural language, formal logic and discourse representation theory*. Studies in linguistics and philosophy, No. 1 (Amsterdam: Kluwer Academic).

[B22] Kanazawa M. Shimada J. (2014). “Toward a logic of cumulative quantification,” in Joint proceedings of the second workshop on natural language and computer science (NLCS’14) and 1st international workshop on natural language services for reasoners (NLSR 2014), Vienna, Austria, July 2014. Editors de Paiva V. Neuper W. Quaresma P. Retoré C. Moss L. S. Saludes J. (Coimbra, Portugal: Centre for Informatics and Systems, University of Coimbra), 111–124.

[B23] Kennedy C. (2007). Vagueness and grammar: the semantics of relative and absolute gradable adjectives. Linguistics Philos. 30, 1–45. 10.1007/s10988-006-9008-0

[B24] Kobele G. M. Zimmermann M. (2012). “Quantification in German,” in Handbook of quantifiers in natural language of Studies in Linguistics and Philosophy. Editors Keenan E. L. Paperno D. (New York, NY: Springer), Vol. 90, 227–283.

[B25] Kratzer A. (2007). “On the plurality of verbs,” in Event structures in linguistic form and interpretation. Editors Dölling J. Heyde-Zybatow T. Schäfer M. (Berlin: Mouton de Gruyter), 269–300.

[B26] Krifka M. (1992). Definite nps aren’t quantifiers. Linguistic Inq. 23, 156–163.

[B27] Krifka M. (1999). “At least some determiners aren’t determiners,” in The semantics/pragmatics interface from different points of view of current research in the semantics/pragmatics interface. Editor Turner K. (Amsterdam: BV Elsevier Science), 1, 257–291.

[B28] Landman Fred. (1996). “Plurality,” in Handbook of contemporary semantics. Editor Lappin S. (London: Blackwell), 425–457.

[B29] Lang E. Carstensen K.-U. Simmons G. (1991). Modeling spatial Knowledge on a linguistic basis: theory—prototype—integration. Lecture Notes in Artificial Intelligence No. 481. Springer

[B30] Lang E. Maienborn Claudia. (2011). “Two-level semantics: semantic form and conceptual structure,” in Semantics. An international handbook of natural language meaning (=HSK 33.1). Editors von Heusinger K. Maienborn C. Portner P. (Berlin, New York: de Gruyter), 709–740.

[B31] Link G. (1983). “The logical analysis of plurals and mass terms: a lattice-theoretical approach,” in Meaning, use and the interpretation of language. Editors Bäuerle R. Schwarze C. von Stechow A. (Berlin, New York: de Gruyter), 303–323.

[B32] McDonald D. D. (1993). Issues in the choice of a source for natural language generation. Comput. Linguistics 19, 191–197.

[B33] Montague R. (1973). “The proper treatment of quantification in ordinary English,” in Approaches to natural language. Editors Hintikka K. J. J. Moravcsik J. M. E. Suppes P. (Dordrecht: Reidel Synthese Library), 49, 221–242.

[B34] Newell A. (1982). The knowledge level. Artif. Intelligence 18, 87–127. 10.1016/0004-3702(82)90012-1

[B35] Nirenburg S. Raskin V. (2004). Ontological semantics. Cambridge: The MIT Press.

[B36] Nouwen R. (2014). “Plurality,” in Cambridge handbook of semantics. Editors Dekker P. Aloni M. (Cambridge University Press), 267–284.

[B37] Nouwen R. (2010). “What’s in a quantifier?,” in The Linguistic Enterprise: from knowledge of language to knowledge in linguistics. Editors Everaert M. Lentz T. de Mulder H. Nilsen O. Zondervan A. (Benjamins: Linguistik Aktuell), 235–256.

[B38] Parsons T. (1995). Thematic relations and arguments. Linguistic Inq. 26, 635–662. 10.1016/0003-6870(95)00039-f

[B39] Pelletier F. J. (2010). “Mass terms: a philosophical introduction,” in Kinds, things, and stuff: mass terms and generics. Editor Pelletier F. J., 123–131.

[B40] Peters S. Westerståhl D. (2006). Quantifiers in language and logic. New York: Oxford University Press.

[B41] Reyle U. (1993). Dealing with ambiguities by underspecification: construction, representation and deduction. J. Semantics 10, 123–179. 10.1093/jos/10.2.123

[B42] Sæbø K. J. (1995). “Quantifiers: configurations and interpretations,” in The Blaubeuren papers. Proceedings of the workshop on recent developments in the theory of natural language semantics. Editors F. Hamm, J. Kalb, and A. v. Stechow, Vol. 2, pp. 347–378. Seminar für Sprachwissenschaft, Tübingen.

[B43] Safir K. Stowell T. (1988). “Binominal each,” in Proceedings of the 18th annual meeting of the north east linguistic society (NELS 18), New Orleans, December 27–30, 1988, 426–450.

[B44] Scha R. (1984). “Distributive, collective and cumulative quantification,” in *Truth, interpretation and information*, Groningen-amsterdam studies in semantics. Editors Groenendijk J. Janssen T. M. V. Stokhof M. (Berlin, Boston: De Gruyter Mouton), 2, 131–158.

[B45] Scha R. Stallard D. (1988). “Multi-level plurals and distributivity,” in Proceedings of the 26th annual meeting on association for computational linguistics ACL ’88, Buffalo, NY, June 7–10, 1988 (Stroudsburg, PA: Association for Computational Linguistics), 17–24.

[B46] Shaw J. McKeown K. (2000). “Generating referring quantified expressions,” in INLG’2000 proceedings of the first international conference on natural language generation, Mitzpe Ramon, Israel, June 12–16, 2000 (Mitzpe Ramon, Israel: Association for Computational Linguistics), 100–107.

[B47] Sher G. (1990). Ways of branching quantifers. Linguist Philos. 13, 393–422. 10.1007/bf00630749

[B48] Steedman M. (2012). Taking Scope. The Natural Semantics of Quantifiers (Cambridge: MIT Press).

[B49] Szabolcsi A. (2010). Quantification. *Research surveys in linguistics* (Cambridge: Cambridge University Press).

[B50] Takac M. Knott A. (2016). “Working memory encoding of events and their participants: a neural network model with applications in sensorimotor processing and sentence generation,” in Proceedings of the 38th annual meeting of the cognitive science society (CogSci), Austin, TX, August 10–13, 2016. 2345–2350.

[B51] Tunstall S. L. (1998). The interpretation of quantifiers: semantics and processing. PhD thesis. Amherst (MA): University of Massachusetts Amherst.

[B52] Winter Y. (2002). Atoms and sets: a characterization of semantic number. Linguistic Inq. 33, 493–505. 10.1162/002438902760168581

[B53] Zimmermann M. (2002). Boys buying two sausages each: on the syntax and semantics of distance-distributivity (Utrecht: LOT).

